# Brain Traumatisms

**Published:** 1900-06

**Authors:** Howard J. Williams

**Affiliations:** Surgeon to Macon Hospital, etc.; Ex-President Medical Association of Georgia, etc., Macon, Ga


ATLANTA

### Journal-Record of Medicine.

Successor to Atlanta Medical and Surgical Journal, Established 1855, and Southern Medical Record, Established 1870.

VOL. II. JUNE, 1900. NO. 3.

EDITORS { BERNARD WOLFF, M.D., DUNBAR ROY, M.D.

M. B. HUTCHINS, M.D., BUSINESS MANAGER.

PUBLISHED MONTHLY. OFFICE 305 AND 306 FITTEN BUILDING.

### *ORIGINAL COMMUNICATIONS.*

### BRAIN TRAUMATISMS.

BY HOWARD J. WILLIAMS, A.M., M.D.,

Surgeon to Macon Hospital, etc.; Ex-President Medical Association of Georgia, etc., Macon, Ga.

No field of modern surgery offers so grand a promise of brilliant and daring operative work as the brain and its envelopes; and none other requires for its successful conduct a broader, deeper knowledge of the functional life of the region involved. The application of the discoveries of cerebral localization to diagnosis has done more to advance surgical intervention in these diseases and accidents than have the lessons of Lister and the aseptic refinement of his discoveries. Notwithstanding this, there is no region of the body in which the significance of symptoms is more delusive, nor in which the results of operation are more problematical. In traumatic lesions is this particularly true. Except in the grosser lesions involving the motor areas particularly, localization of disturbed cerebral functions has generally been of but little practical value in indicating the point of the brain most importantly involved. The reasons for this are:

First: In the larger majority of brain traumatisms multiple lesions exist, many special functions are involved and conflicting symptoms appear.

Secondly: The general symptoms of cerebral shock (or concussion, if you will) and compression usually so cloak the symptoms pointing to special regions involved, that important lesions cannot be discovered.

Thirdly: The general depression common to various lesions may be so prolonged, that, when it is relieved, the symptoms pointing to special obtunded functions may be discovered too late to be of working value, secondary changes having destroyed the cells of the particular regions involved.

Lastly: While in some regions of the brain a cerebral function is circumscribed to a particular small seat, in other parts it is diffused over large areas; here compensation of the obstructed functions is early taken up by unaffected cells, the functions are restored, and the significant symptoms have disappeared before the general depression has passed off; partial loss of power of the part affected may later become permanent when secondary changes have destroyed the involved area. To make cerebral localizing symptoms of working value, their study should begin with the beginning of the case, watching for their earliest manifestations in the midst of the symptoms of the general traumatism.

Too much attention has, heretofore, been devoted to the study of the incidents of brain traumatisms, the so-called concussion of the brain, the general compression symptoms, and the character and location of fractured bones of the skull. The time is now present in advanced surgical diagnosis when the location of special pressure symptoms of localized hemorrhages and edemas should be studied in the earliest hours of head injuries; and the irritation symptoms of localized brain lacerations looked for while symptoms of gross hemorrhage, depressed bone, or signs of fracture of the base of the skull exist. Why should the once important condition, concussion of the brain, occupy the surgeon's attention and divert his efforts from locating and relieving the more important lesions following traumatisms? This condition is now recognized as being nothing more nor less than surgical shock, and is identical with that condition present when there is traumatism elsewhere in the body. The same class of symptoms and signs is produced by a blow over the heart or on the solar plexus. Men die of shock following blows in these regions, just as they die of concussion following traumatisms of the head, and the same absence of visible signs is found on autopsy as often exist after death in concussion. Cerebral concussion has been the death-warrant of many cases having remediable lesions. The term is a subterfuge of ignorance and is an excuse for timorous non-interference.

Again our ignorance of or confusion of ideas relative to the condition recognized as compression has often led us to overlook a pathological condition existing in the brain tissue, causing prolonged unconsciousness, and other grave symptoms, which we attribute to a depressed bone or an insignificant hemorrhage. We fail to see the signs of localized laceration of the brain tissue, while relieving the general symptoms of compression by elevation of bone or turning out clots.

We lay too much stress on the presence of fracture of the skull in head injuries. Fracture, either of the vault or base, is *per se* of comparatively little moment, and yet how often do we hear and read of fractures of the skull causing unconsciousness, disorders of pulse and respiration, temperature variations, and other undoubted symptoms and signs of serious intracranial injuries. Depression, uncomplicated by cerebral lesion, is almost never a cause of early symptoms; compound fracture is only of danger in that it is an open avenue for infection; and the signs of fracture at the base are only of importance in that they show the location of important lesions of the contents of the skull.

It is with these conceptions of popular fallacies that the writer undertakes to present the following nine cases; and, together with a review of a table of one hundred and twenty-six reported cases hastily gleaned from a limited number of medical periodicals, to show that in all serious traumatisms of the head there exist at some point in the brain or its membranes lesions of greater importance than is concussion, compression, or fractured bone. These are the mere incidents of the graver intracranial hemorrhages, contusions and lacerations of the brain or its membranes; either of which intracranial lesions is always present individually in severe head injuries, or in combination with each other.

The study of the symptomatology of cerebral traumatisms will soon convince one of the fact that the symptoms are dependent directly upon interference with functions, and can scarcely be attributed to the violence itself, to the concomitant lesion of the bony covering, to the ruptured blood-vessel, or escaped blood, or to the contusion or laceration of the brain tissue *per se*. In other words, as Gowers says, "The symptoms depend immediately on the changes in the nervous elements and are secondary to what we call the disease." For instance, a blow on the head fractures the skull, producing a rupture of the middle meningeal artery, causes a clot to press upon the motor area of the brain; the paralysis of the opposite side of the body is directly due to the changes in the nervous elements resulting from the arrest of the nutrition of the region pressed upon. The symptoms, therefore, are not directly dependent upon either the blow, the fracture, or the hemorrhage, but on the results of the localized anemia.

The symptoms of brain traumatisms are general and special. General symptoms are those common to all injuries or diseases of the brain—coma, convulsions, delirium, headache, vertigo, insomnia, mental irritability and vomiting. They are usually due to the increase of the intracranial contents by hemorrhage, serous effusion or pus. The special symptoms depend directly for their production upon irritation or destruction of particular parts of the brain; disturbance of motion, paralysis; disturbances of sensation, anesthesia; disorders of special senses, sight, hearing, etc.; defects of memory and loss of speech, aphasia. Acute lesions produce symptoms severer than correspond with the damage actually done, due to the attendant shock and physical depression; hence the general symptoms are at first often misleading, but as time passes and restoration advances, the true estimate of the damage is more apparent and the exact seat may be discovered by the direct or focal symptoms. It is necessary then to be on the alert for the special direct symptoms, as they appear while the general symptoms are disappearing.

Lesions of bones have but little to do with the conditions existing within the skull; no more or no less than the injuries of the abdominal walls have to do with lesions of the abdominal viscera. The relation between a contused scalp, with a fracture of the skull, to the lacerated brain within, is analogous to the relation of a severe contused wound of the abdominal wall to the laceration of the contained viscera, following a blow or a kick; it hides from view the greater injury. By far the most serious and dangerous brain traumatisms are those which occur either without fracture or with fissured or stellated solutions of the continuity of the skull. In my table of 126 cases there are reported 49 cases of hemorrhages, lacerations and contusions without any fracture of the skull, and 22 cases of clots, lacerated, contused and disorganized brain-matter present with fissured fracture alone; while these lesions were present with all the other forms of fracture except in four cases. The greatest mortality was in these two classes. In an experience of twenty years the writer recalls only one case of a compound depressed fracture without additional lesion of the brain-matter or its membranes. In the table, as has just been said, there were two cases of uncomplicated depressed fracture, and two of uncomplicated compound depressed fracture. These uncomplicated fractures are by far the simplest cranial injuries that we have to contend with if promptly recognized and properly treated. In proof of this there was complete recovery in the four cases mentioned, without any early serious cerebral symptoms; they recovered without operation. Whether there was evidence of irritation later in life, the writer is unable to say, because the reports were only of the early condition.

The following is the report of the case recalled by the writer:

CASE 1.—*Absence of Brain Lesion and Symptoms; Compound Fracture with Depression of Bone; Trephining; Recovery.* S. C., adult negro laborer, was struck with an iron bar on the right parietal eminence, October 12, 1889. He was stunned for a few minutes, but got up and walked a distance of one mile to my house.

There was present a longitudinal cut of the scalp three inches long, exposing a depressed fracture. Temporary dressings were applied and he went home. Two hours later there were no signs of compression or of laceration of the brain, no unconsciousness, no paralysis, nor muscular twitching. The pupils were equally responsive to light. Pulse 80, respiration 28, temperature 99.6. The writer trephined and elevated the broken piece of bone, which was depressed half an inch at the edge. There was no clot and the brain pulsated freely in the trephine wound.

In two weeks, without intervening symptoms, he was completely recovered.

A compound fracture, when complicated by cerebral injuries, bears the same relation to the lacerated brain or hemorrhagic effusion that a penetrating wound of the abdominal walls bears to an injured viscera. It is only a complication by being a possible avenue of infection, and when undepressed should receive the same treatment. In no other way does it add to the dangers of the case. In the table there are five cases of secondary infection, dependent upon the attendant compound injury of the bone. The next case presented bears out strongly this point in my argument.

CASE 2.—*Laceration of the Brain; Escape of Brain Tissue, without Early Symptoms; Compound Depressed Fracture; Abscess Tenth Day; Elevation and Drainage; Death.* S. J., white, cattle trader, aged fifty years, July 24th, 1894, in a drunken fight was struck on the left temporal region with the jaws of a closed pocketknife, inflicting a compound depressed fracture one inch above the external auditory canal, from which escaped some brain tissue and copious bleeding. No hemorrhage from ears or nose. Though drunk at the time, he did not lose consciousness.

Six hours later, when seen by the writer, he was still conscious, walking about the house; dull, however, from the whisky which he continued to freely drink; speech slow, but intelligible: urine and feces passed voluntarily; pulse 70, respiration 17, temperature 98.4. The wound, *which had been probed by his physician*, and had no dressing applied, was still bleeding, but not so freely as immediately after the fight. He and his family declined to have any interference except an antiseptic dressing.

His condition remained unchanged, except a slight rise of temperature on third day (99.4), a hesitancy of speech and occasional "stupid spells" at night, until the tenth day.

August 2d, 7 P.M., he was seen for the third time. He had aphasia without other paralysis; was stupid, irritable, restless; urine passed involuntarily; left pupil contracted and stationary; right normal; pulse 56, full; respiration 14, irregular; temperature 98. No history of a chill or convulsions, but an increasing restlessness and dullness of mind for the past three or four days. An operation was advised, but not permitted until the next afternoon, when he was profoundly unconscious.

The wound was enlarged and the bone elevated. There was an escape of some blood and about two drachms of pus. The wound was drained and an antiseptic dressing applied. He never fully regained consciousness; pulse rose to 120, respiration 32, temperature 102. He died twenty-four hours after the operation.

Hemorrhage and clot is the most frequent injury found where there are no fractures and in fissured or depressed fractures. It is the most frequent single intracranial injury that occurs, but it is more often found combined with lacerations or contusions. In the table referred to there are twenty-eight uncomplicated hemorrhages, that is, uncomplicated with laceration, or contusion without fracture; twenty-six cases of uncomplicated hemorrhage present with fracture; twelve cases of combined clots, lacerations and contusions existing without fracture; and twenty-two cases of combined laceration and hemorrhage present with fracture. This traumatic lesion of the head is the most easily diagnosticated injury we meet, and yet it was attended by a large fatality, owing to its concealed presence, before cerebral localization of functions made it a matter easily to be recognized, and before prompt aseptic operative treatment instituted. Wiseman gives statistics of two hundred and fifty-seven cases; one hundred and forty-seven treated expectantly, of which one hundred and thirty-one died; one hundred and ten operated upon, of which only thirty died. Of all classes in my table, fifty-eight recovered and twenty-five died. Very few died after operation. Modern treatment of wounds has materially reduced the mortality of this lesion.

That the presence of a fracture is of no moment either in diagnosis, prognosis or treatment of this lesion is apparent when it is known that it occurs as often, if not oftener, in the absence of fracture, and in very trivial as well as violent accidents; and that the two diagnostic points, the presence of a conscious interval, and dilatation of the pupil when the clot is in the middle fossa of the corresponding side, are oftener present in the absence of fracture. The presence of hemiplegia, paraplegia and rigidity occur equally with or without fracture.

The more important point is the early recognition of the presence of an intracranial complication, laceration or contusion, which modifies the lesion and its diagnosis, and which has so frequently caused the confusion between this lesion and the so-called concussion and compression of the brain.

The most important point, however, after ascertaining the possible presence of hemorrhage, is the location of the clot and ascertaining the involved vessel. The most frequent vessel involved in these hemorrhages is the middle meningeal artery; the sinuses are sometimes involved where there is violent injury to the bone. The most frequent seat of the clot is between the skull and the dura. Sub-dural hemorrhages, however, most frequently occur in complicated cases. Cortical hemorrhages complicate laceration of the brain.

Illustrating the comparative unimportance of a comminuted fracture of the skull, in the presence of an epidural hemorrhage, case third is reported. Unfortunately the interval of consciousness in this case was not known when the patient was first seen, although he subsequently recalled events which indicated that it had been present.

CASE 3.—*Epidural Hemorrhage; Comminuted Fracture of the Right Side of the Skull; Evacuation of Clot; Drainage; Recovery.*—S. D., negro tramp, aged twenty-one years, was found February 19, 1898, on the right of way of a railroad near Macon, in an unconscious condition. From the records of the railroad office the accident was supposed to have occurred about 3 A.M. At 8 A.M. he was brought into the hospital. His right clavicle was broken, he was bruised about the body, the right eye bulged slightly; the right side of the head was flattened and the overlying scalp was boggy, but not torn. There was no hemorrhage from the ears or nose. He was semi-comatose, would answer incoherently, and was constantly tossing. The left leg was alone paralyzed, the left arm feebly moved, and the face was twitching. Pupils equally dilated and responsive, the right eye turned up and to the right. Pulse 74, respiration 26, labored; temperature 98. Vomited once, and his urine had to be drawn.

At 9 A.M. his scalp was opened. Periosteum had been detached from the skull, and there was considerable blood under the scalp. The frontal, temporal and parietal bones were greatly broken up; one piece, one and a half by two and a half inches, involving the parietal, was removed; the other parts were raised. A clot found between the skull and dura was removed. The scalp was sewed up, a gauze drain being left in the most dependent point of the incision. Three hours later he told his name and residence and could move his left leg. For the next three days he rapidly improved, although he had to be catheterized and had an evening temperature of 100 degrees. The fourth day he recalled the events of the night of the accident sufficiently clearly to indicate that there had been an interval of consciousness. On the twenty-first day he was discharged, completely recovered.

Laceration and contusion of the brain are by far the most important injuries in these traumatisms. These grosser lesions of the brain are identical with contusions and lacerations elsewhere in the body, and the clinical history corresponds with what we should naturally expect from a lesion of such delicate tissue and specialized functions. They are the lesions that give rise to the most confusion, and upon which are so often based the diagnosis, "concussion and compression of the brain." Contusion exists to some extent in all head injuries. As an isolated lesion, it is infrequent. Lacerations are less frequent than contusions, and rarely exist alone uncombined. These lesions exist whether the skull is fractured or not, and apparently the most marked depression and symptoms occur in those cases unattended by destructive lesions of the bones. In fact, it has seemed to the writer that in those cases where there was extensive compound fracture of bone and laceration of the membranes with escape of brain matter, there was a better outlook for the final recovery of the patient. In the table there were only nine cases of uncomplicated laceration and contusion, twenty-five cases of laceration attended by fracture, and twenty-two cases of clots and laceration present with fracture.

The symptoms of these lesions, continuous unconsciousness, delirium, headache, irritability, vertigo, convulsions, loss of sphincteric control, bilateral inequalities of pulse and bilateral irregularities of temperature—for a long time considered concussion and compression symptoms, dependent upon the so-called vibratory action of the encephalic nerve cells and cerebro-spinal fluids—occur independently of fracture, and can in no way be attributed to any form of lesion of the skull. The destruction of the functions of the special senses—sight, hearing, speech and smell—so frequently present in these lesions, and valuable as focal symptoms, occur independently of lesions of the bone. Depressed and compound fractures have no influence whatever on the special direct symptomatology of these lesions.

The fourth case is introduced as a fair representative of severe contusion of the brain, complicated by laceration of the longitudinal sinus, occurring in the presence of a severe bone lesion which had no direct bearing on the symptomatology.

CASE 4.—*Severe Contusion of the Brain, and Laceration of the Longitudinal Sinus; Compound Depressed Fracture; Elevation; Death.* D. J., white, aged thirteen years, was kicked, September 27, 1891, on the vertex by a horse. He was taken up unconscious and remained so for three hours. He then could answer questions intelligently for a short time, but lapsed into a semi-comatose condition. Delirium and irritability were soon present.

Twenty-four hours later, when the writer saw him, there was a constant slow oozing of blood from an irregular scalp wound on top of the head, through which a depressed angle of bone could be felt to the right of the sagittal suture. No hemorrhage from the ear or nose, nor any ecchymosis about the eyes. He would answer irritably when urged to do so, and was generally stupid. The left foot and leg were paralyzed; the left arm and face twitching. Pupils equally contracted. Pulse feeble, 120; respiration 26, sighing; temperature normal. He had vomited several times and passed urine and feces involuntarily.

The scalp was raised and the depressed pieces of the skull, covering an area two by three inches, were elevated. While removing an irregular piece of bone, which projected below the sagittal suture, a tremendous venous hemorrhage took place, which was with difficulty arrested by packing the wound with gauze. The longitudinal sinus had been irregularly torn. The dura was also torn at the same point, and, thinking the paralysis was possibly due to a subdural clot, this wound was enlarged. No clot was present, but the cortex of the brain was badly contused and softened. The dura was closed, a drain of gauze was brought out of the lower angle of the scalp, which was sewed up, and aseptic dressings applied.

The lad never regained consciousness after the operation, and eighteen hours later died.

Case fifth is introduced illustrating laceration of the brain, complicated by epidural hemorrhage, present with fracture of the skull.

CASE 5.—*Laceration of the Brain, and Epidural Hemorrhage, with Extensive Fracture of the Vertex and Base of the Skull; Trephining; Death.* J. S., adult negro, track hand, fell, October 15, 1890, under a crank car, the axle striking his head. Unconscious at once. Was brought to Macon and seen by the writer two hours later. There was hemorrhage from the left ear and nose, left eye bulged out, scalp edematous; bones under scalp felt irregularly displaced. Still unconscious; right arm and leg partially paralyzed, left moving in clonic spasms; said to have had two convulsions on the way up to the city. Pupils dilated, irresponsive to light. Pulse 52, hard, irregular; respiration 14, irregular and stertorous; temperature 101. Involuntary passage of urine.

Scalp opened and the frontal, parietal, and occipital bones found comminuted. The trephine was applied to raise the bone. A small clot was found over the left motor area and removed. The dura was torn over the frontal lobe, and some brain matter exuded through the rent. Rent in dura closed partially and scalp closed, with gauze drainage.

October 16. Pulse 88 to 116, respiration 40 to 44, temperature 100 to 101.4. Stuporous, but answers questions connectedly; moves right arm and leg freely, muscular twitching still present; irritable and restless; pupils contracted but respond; bladder and bowels act involuntarily; expectorates bloody mucus.

October 17. Mind rational; muscular twitching relieved; intense headache; recovered control of urine and feces. Pulse 98, respiration 26, temperature 100.

October 18. Delirious, then unconscious; twitching of face and arms. Pulse 144, respiration 40, temperature rapidly rising, 104.2 in the evening.

October 19. Coma, temperature 105; death 3 A.M.

The next two cases are of uncomplicated contusion and laceration, and well illustrate the history of such lesions.

CASE 6.—*Probable Contusion of the Brain, without Detectable Fracture of the Skull; Recovery.* T. S., negro brakeman, aged thirty-one years; was struck, February 22, 1899, on the back of the head by an overhead bridge, and precipitated to the ground. There was no evidence of fracture of the skull discovered through the scalp; the scalp was severely bruised and swollen. There was no hemorrhage from the nose or ears. He was completely insensible from the first and remained so for several hours. No paralysis was present. In twenty-four hours he was sent to Macon. He could then walk with assistance, but held his head rigidly erect; face and arms at times twitched. Had vertigo, intense headache; was dull and restless. Pupils normal; vision unaffected. Pulse 86, respiration 28; temperature, right axilla, 100; left 99.6. Vomited on the way home; urine and feces controlled.

March 1. Mental dullness increased; at times extremely irritable, or again emotional; pulse 90, respiration 26, temperature, right axilla 99.2, left 99 at morning visit, but at the evening visit it was 98.7 in both axillæ.

March 2. Delirious, uncontrollable at times; would get out of bed and scream with pain in the back of the head. Was sent to the hospital, where he remained four days; delirious at night, clear mentally during the day, but restless, irritable and emotional; persistent occipital headache; vision perfect, pulse varying between

84 and 96, respiration 16 to 26, temperature 98.4 to 99; begged to go home so earnestly that he was allowed to do so.

During the next three days there was some improvement in mental condition, but his muscular motions were slow and stiff.

March 9. Hard to control, irritable, pugnacious; occipital pain and vertigo constantly present; slow, stiff, reeling gait.

March 12. Almost complete imbecility; cries and mopes about the house and yard; headache; slight irregular rise in temperature.

From this date there was slow improvement; mind acting slowly, muscular action stiff and difficult.

May 1. Recovery complete.

CASE 7.—*Probable Laceration of the Brain; Fracture of the Frontal Sinus; Recovery.* S. W., negro brakeman, twenty-five years of age, July 6, 1899, was missed from his train a few minutes after leaving a meeting point, and the section master notified to look for him. He was found between the tracks at the meeting point in an unconscious condition and bleeding freely from a wound of the right frontal sinus, which was crushed in by some irregular body. There was hemorrhage from the nose and ecchymosis of the right eyelids. When admitted to the hospital, sixteen hours after the accident, unconsciousness was still complete; no paralysis was present, but there was jactitation of the limbs. Pupils contracted, but responsive. Pulse 72, respiration 24, temperature 100. Urine involuntary.

The wound was enlarged without anesthesia, and it was ascertained that there was no fracture of the inner walls of the frontal sinus.

Unconsciousness gradually passed off in twenty-four hours, giving way to irritability and mental dullness; occasional twitching of the face; urinary incontinence still present. Temperature 100, respiration 19, pulse 54. There was a marked difference in the volume of the two radial pulses, that of the left being feeble, while the right was strong and full.

The following nine days his mental condition was variable, having dullness, volubility, hallucinations and persistent frontal headache. The temperature varied between 99 and 100; urinary control returned after the third day. He could not recall the incidents of the accident until the third week. He said he remembered the engine of the passing train catching his coat and his falling on a cross-tie. Recovery was complete in four weeks.

We take up next the question of injuries of the brain at the base, that class of cases which are misnamed "fractures at the base." They are injuries of the brain proper—hemorrhage, contusion and laceration—the evidences of fracture being of value only as indicating the location of the brain injury. These lesions are most often accompaniad by evidences of a high degree of edema and increase of the intracranial pressure; coma complete, without evidences of general paralysis, symptoms of graver lesions than bone fracture and of deeper significance.

Fractures in this region are usually linear or fissured, and extend to the base from the point of impact of the vulnerating force on the vertex or other portions of the skull, reaching the base by the shortest anatomical route, traversing the natural fissures in their course. The signs of these fractures are ecchymosis about the mastoid, occipital region, eyelids and conjunctiva; hemorrhage from the ears, nose and mouth, followed by escape of cerebro-spinal fluid; paralysis of a single nerve at the base may occur when the foramen is fractured and the nerve is lacerated by the bone. Escape of cerebro-spinal fluid is not, however, always pathognomonic of fracture. It may escape through a natural foramen leading from the inside of the skull, if the membranes have been lacerated without a fracture. A great bulk of the hemorrhage always comes from the vessel of the arachnopial membrane and the temporosphenoidal fissure, passing along the foramen of the auditory nerve in the petrous portion of the bone.

The focal symptoms of injury at the base are symptoms of irritation or destruction of the basalar regions of the brain and of the cranial nerves as they traverse the cerebral cavity to their exits. There may be loss of sense of smell, one-sided blindness, half vision, ptosis, dilated pupil, external strabismus, loss of accommodation, paralysis of movement of the eyeball, unilateral paralysis of sensation of the face and one-sided paralysis of the face. If it is a simple irritative lesion, the paralyses pass away; if the nerves are lacerated, it is permanent. The facial nerve may be paralyzed in the aqueduct of Fallopius by indirect fracture, the injury being permanent, the palate escaping. If the auditory nerve is permanently affected, the petrous bone is broken across, or it may be due to an unabsorbed hemorrhage into the tympanic cavity, without fracture of the base.

With these exceptions the bone lesions have no influence on the focal signs and symptoms, and they are present independently of the bone injury.

The fatality of these cases is due directly to the injury of the brain, and is most commonly due to infection traveling through the new opening in the bone. The fatality in preantiseptic days was greatly in excess of that at the present time, and, excluding the extra risk of sepsis, the prognosis is on the whole better when there is escape of cerebro-spinal fluid than without it; the intracranial pressure being relieved by the continuous drain of this fluid. Of the 126 cases in the table, there were 13 basal injuries, with 9 recoveries and four deaths. Case 60 of the table is a good illustration of injury of the base of the brain, together with a permanent facial paralysis due to a fracture of the Fallopian canal. The following case also illustrates the permanent paralysis of the facial nerve, dependent upon an indirect fracture of the external auditory canal, the facial nerve being involved internally to the chorda tympani in the aqueductus Fallopii; the temporary paralysis of other nerves was due to intracranial injuries.

CASE 8.—*Laceration or Contusion of the Brain; Fracture of the Base of the Skull; Recovery.* Wm. M., sailor, adult, white, December 15, 1881, fell fifteen feet on his right ear and mastoid process. When admitted to the Jefferson Medical College Hospital (at which institution the writer was then an interne) four hours after the accident, there were puffiness of scalp about the ear and hemorrhage from the right ear, with several hours later subconjunctival and orbital extravasation, and ecchymosis over the right mastoid bone. He was unconscious immediately after the injury and remained so twenty-four hours. Pupils were contracted, but responded to light; eyes directed up and to the right; pulse 66, full, respiration 26, temperature 101, five hours after the accident.

Twenty-four hours later: Coma passed into stupor; could answer questions when aroused; refused to swallow; paralysis of the right side of the face, and right-sided ptosis; anesthesia of right side of face; muscular twitching of the upper limbs, irritable when aroused; urine and feces passed involuntarily; pulse 72, respiration 22, temperature had declined from 102.6 of the previous night to 99.

In four days the anesthesia of the face had passed, except of the eyeball; facial paralysis and ptosis were still present; persistent pain in the right side of the head; speech was hesitating; the mind was clear, but he was irritable and emotional, crying without any excitement. The control of the bowels and bladder had returned. Pulse and respiration were normal, but the temperature remained 99 to 99.4.

He gradually recovered in two months; the paralysis of the right side of the face, right side palatine muscles, and ptosis persisted. The sense of taste was lost on the right side of the tongue. Right sided headache and emotional disturbances were still present.

The next case presents a group of focal symptoms of laceration of the brain at the base and a late complication due to morphological changes of a hemorrhagic deposit in a lacerated region.

CASE 9.—*Laceration of the Brain; Traumatic Cyst of the Third Frontal Convolution; Fissured Fracture of the Frontal and Temporal Bones, Extending to the Base; Late Trephining and Evacuation; Recovery Incomplete.* Mrs. E. J. C., December 2, 1899, was thrown from her trap and taken up immediately, unconscious. The right humerus was broken near the head of the bone. There was also a small lacerated wound at the external angle of the right supraorbital process, and limited contusion of the same region. The right eyeball was protruding and turned up and outwards. Ecchymosis of the right conjunctiva; and hemorrhage in the orbital fossa, from the nose, and slightly from the left ear. The left arm and both legs moved freely; muscular twitching of the face and arms; apparent perception of local irritation; involuntary urination; groaning and stertor; pupils dilated, but responsive; pulse 78, irregular and intermittent, respiration 28, temperature 99.4, three hours after the accident. Vomited decomposed blood six hours after the injury.

On the sixth day the writer was first asked to see her. The unconsciousness, restlessness, involuntary evacuations, pulse, respiration and temperature were unchanged. On this day it was found that she had loss of sensation of the right conjunctiva and side of face, with a squint and ptosis of the right eye. The discharge from the ear had ceased the fourth day.

Seventh day. Paralysis of the right side of the face; consciousness returning, she could answer "yes," and recognize friends; could move some in bed. Involuntary fecal and urinary actions persisted; pupils normal, pulse 58 to 72, respiration 16 to 20, temperature 98 to 99.6.

From the eighth to the thirty-ninth day her mental and physical condition was very variable, either wildly delirious, restless, or quietly sleeping and having a few moments of lucidity. Took liquid foods mechanically; gradual emaciation. The anesthesia, facial paralysis and ptosis had gradually passed off. Involuntary fecal and urinary action persisted, except a few times near the end of this interval. The temperature was usually subnormal and rarely more than 99.5. The advisability of operative measures was fully and frequently discussed.

At 9 P. M. of the thirty-ninth day she had a chill with a temperature of 103, pulse 120, extremely weak and irregular, respiration 44. Cyanosis and great distress attended this condition; pupils widely dilated.

The next day the writer, not having seen her for several days, discovered a tender spot just over and in front of the right ear and in an area of pre-existing bogginess of the scalp. The temperature had gradually declined to 99.2, with an attendant reduction of the pulse and respiration. The usual variableness of her mental condition was present.

There was a second sudden rise of the temperature (101.2), pulse (100) and respiration (28) in the early morning of the forty-third day, with extreme restlessness, difficult deglutition and labored breathing. In the evening she was profoundly unconscious; the nurse thought she was dying during the night. An operation was advised, and Dr. McRae of Atlanta came down in consultation.

January 14. Dr. McRae and the writer, assisted by Dr. Derry and the attending physician, Dr. Holt, trephined the skull over the right ear in the area of tenderness above mentioned. The skin and periosteum being turned down, a fissured fracture was found outlined across the frontal and temporal bones, starting in the region of the external angle of the supra-orbital ridge and passing backwards and downwards behind the external auditory canal. The dura being exposed, it did not pulsate. The dura was then incised about one inch above and to the front of the auditory canal; no subdural clot was found. The brain beneath the incised wound was punctured with a tenotome, and at about an eighth of an inch depth the knife passed into a cavity. The groove director followed the tenotome, and there was a flow of about three drachms of clear liquid from this cavity. The brain tissue, which was of rather pale yellow color, collapsed after the escape of the fluid; the brain then began to pulsate underneath the dura. The incision in the dura was closed with catgut, the scalp wound closed, an epidural drain of gauze being placed in the lower angle of the wound; the usual antiseptic dressing was applied and the patient returned to bed.

After the operation the pulse, respiration and temperature rose and continued high for six or seven days. Unconsciousness was complete for the same interval, while the pupils were not materially affected. A difference between the two axillary temperatures began the first day after the operation, and gradually widened until the early morning of the third day, then gradually returned to equal registration on the fourth day. The right axillary temperature was the higher, and the widest difference was 1.2 degrees. The rectal temperature was higher than the highest axillary temperature. If there was any axillary inequality in the first days of the brain injury, it was not recorded in the notes of the case.

Complete primary union followed the operation, with a continuous improvement in the physical condition of the patient, but no material benefit to her mental life resulted. She is now in a condition of chronic dementia.*

Sphincteric loss was marked in this case, and the difference in the axillary temperature is the first that the writer has observed

---

* Since the above was written this patient has been again trephined. in Philadelphia, by Dr. W. W. Keen, and softening of the right frontal lobe demonstrated.

after operative procedure, but illustrates the fact that any irritation of the brain may bring about this condition.

This case is one of remarkable interest, presenting as it does so many of the symptoms due to injury at the base of the brain, so clearly locating the points of injury and the unfortunate results of failure to relieve early injury. The writer believes that if, as soon as the basal symptoms appeared, an opening had been made and drainage allowed, much if not all of her troubles would have been relieved. The secondary changes, cystic degeneration, softening and nerve-cell destruction may possibly have been prevented.

It is the writer's conviction that more can be done in these cases by early interference than has been done up to the present time. Drainage of the epidural region, or even of the arachnopial cavities, would relieve the intracranial pressure and restore the nutrition of the compressed brain. And, further, the removal of the disorganized brain tissue may be necessary. This is forcibly impressed upon the mind by the records of three cases in the table, where there was a free opening and escape of contused brain matter, blood-clots and cerebro-spinal fluid, with recovery.

Cranial openings should not be different from an opening in abdominal surgery under aseptic rules. In Case 9 the patient was relieved of impending death late in the disease by cranial opening, evacuation of the cyst and drainage.

Nor is the writer alone in the belief that such procedure may be of value. Stimson, Markoe, Fisk, and McCosh report cases (Annals of Surgery, March, 1899), and believe that there is a future for this kind of work. For diagnostic reasons the cranial cavity should be entered as readily as is the abdomen. The lessons of drainage in laceration of the brain, with hemorrhage, attended by extensively compound fractures, should not be overlooked. In cases of slight rise of temperature, prolonged unconsciousness, restlessness, wild delirium and focal symptoms, the question of operative interference arises. If, after a severe blow, there be localized contusion and congestion, with a sub-arachnoid serous exudation, the fluid being imprisoned and symptoms of focal paralysis follow, relief to such conditions may be brought about by free incision and drainage, as it is elsewhere in the body by the same measures.

